# Extremely Low Birthweight Infant with Wolf-Hirschhorn Syndrome: A Dilemma in Determination of the Optimal Timing of Delivery

**DOI:** 10.4137/ccrep.s760

**Published:** 2008-05-16

**Authors:** Shigeo Iijima, Takehiko Ohzeki

**Affiliations:** Department of Pediatrics, Hamamatsu University School of Medicine.

**Keywords:** Wolf-Hirschhorn syndrome, extremely low birthweight infant, retinopathy of prematurity, periventricular leukomalacia

## Abstract

Wolf-Hirschhorn syndrome (WHS) is characterized by multiple malformations as well as mental and developmental defects resulting from the absence of a distal segment of the short arm of chromosome 4. We experienced an extremely low birthweight infant with WHS. The male infant (birthweight 934 g) was born at 31 weeks’ gestation by cesarean section due to intrauterine growth restriction and presented with the typical WHS phenotype. Chromosomal analysis showed a deletion: 46,XY,del(4)(p15.3 p16). Although the patient’s respiratory distress syndrome resolved favourably and his subsequent condition was also stable, he had unusually severe retinopathy of prematurity and periventricular leukomalacia. We suppose that these severe complications were associated with not only prematurity but also with latent structural fragility due to WHS. Herein, we discuss the prenatal detection of WHS and the optimal timing of delivery.

## Introduction

Wolf-Hirschhorn syndrome (WHS) is a chromosomal deletion syndrome with a well delineated phenotype and occurs in 1/50,000 live births. It is caused by a variably-sized deletion of the distal portion of the short arm of chromosome 4 involving band 4p16.3 (WHS critical region: WHSCR). Currently, a new critical region was mapped distally to the WHSCR and designated WHSCR-2 (Zollino et al. 2003). Typically affected individuals have growth retardation, feeding difficulties, developmental delay, and often epilepsy. They have a characteristic facial appearance with prominent glabella, hypertelorism, broad-beaked nose, and frontal bossing, collectively described as “Greek warrior helmet” facies. Congenital anomalies include midline defects (cleft lip/palate, hypospadias, and scalp defects), congenital heart disease, renal and ophthalmic anomalies and skeletal abnormalities (Battaglia et al. 2006). A typical prenatal presentation of WHS is intrauterine growth retardation (IUGR), and all individuals with WHS are born at term and are small for gestational age (Battaglia et al. 2001). We experienced a pre-term, extremely low birthweight infant with WHS.

## Case Report

**Figure 1:**
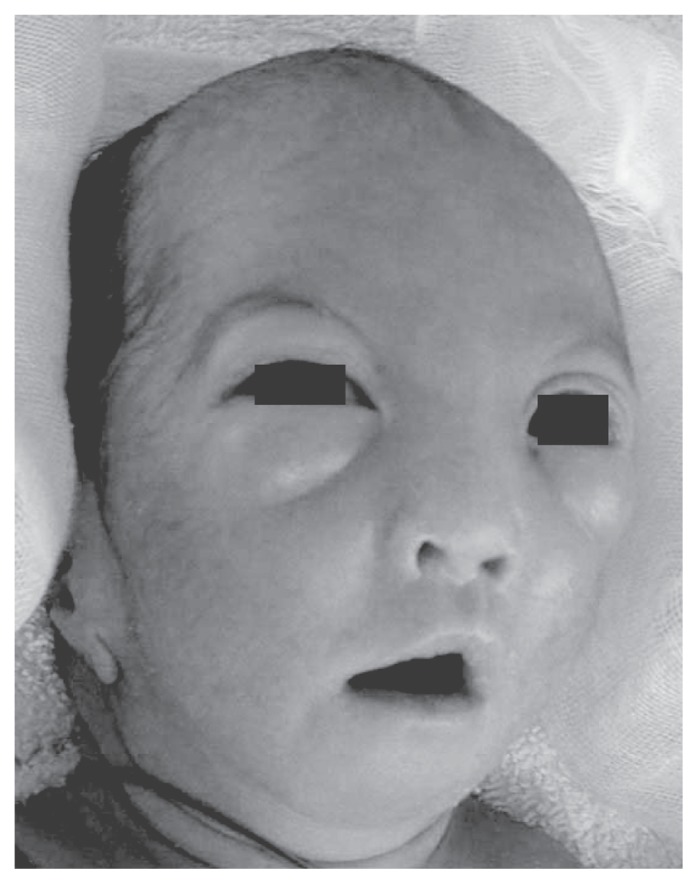
Clinical photograph of the patient at birth.

**Figure 2:**
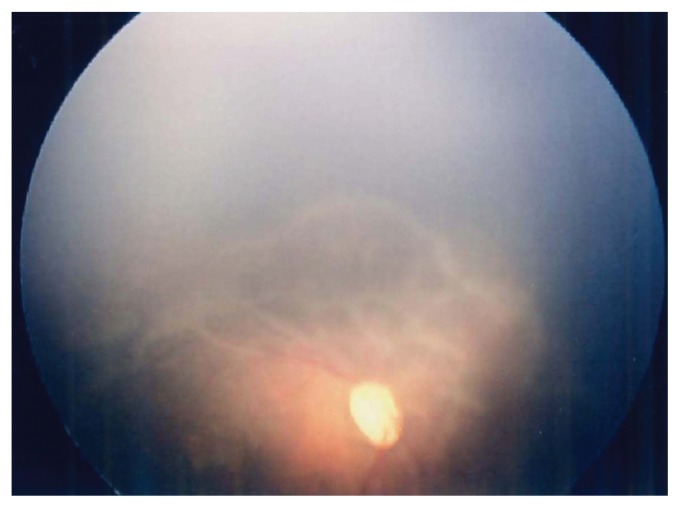
Fundus image of the right eye at 57 days after birth. Note the wide avascular retina with markedly progressed tractional changes.

**Figure 3:**
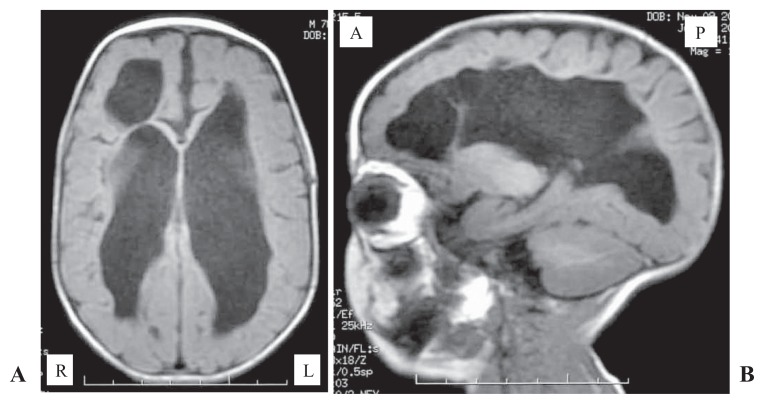
Brain magnetic resonance imaging performed on day 217. **A:** Axial T_1_-weighted section showing enlargement of lateral ventricles with a frontal large cyst. **B:** Parasagittal T_1_-weighted section showing a distinct enlargement of lateral ventricle and global reduction of white matter volume.

The male infant was born to a 27-year-old gravida 1, para 1 woman by cesarean section performed at the gestational age of 31 weeks due to the absence of fetal growth. The pregnancy had been uncomplicated until IUGR was apparent during and after the 26th week of pregnancy. Prenatal ultrasonography (US) and magnetic resonance imaging (MRI) showed no anatomical abnormality, and amniotic fluid volume was normal. Fetal karyotyping was not performed. At birth the weight was 934 g (−3 SD), length 34.5 cm (−2 SD), and head circumference 25.5 cm (−1.5 SD). Apgar scores were 5 at 1 min and 6 at 5 min. In spite of antenatal corticosteroid administration, the infant was intubated after birth and placed on mechanical ventilation for respiratory failure. The placenta (weight 240 g) was delivered intact, and macro- and microscopic examinations revealed no pathology such as malformations, infarction, or chorioamnionitis. Chest radiographs confirmed the initial diagnosis of respiratory distress syndrome, and surfactant treatment was administered. Clinical manifestations disclosed dysmorphic features including high forehead, large rectangular nose continuing to the eyebrows, hypertelorism, low-set ears and carp-shaped mouth (Fig. 1). Postnatal US revealed an atrial septal defect and bilateral renal hypoplasia. He responded promptly to the surfactant treatment, the ventilatory course was favourable, and he was weaned from the ventilator by 9 days of age. His subsequent condition, including the cardiorespiratory state, was generally stable, but weight gain continued to be retarded despite adequate nutritional intake. The first ophthalmic examination was performed at 4 weeks after birth, and the initial signs of aggressive posterior retinopathy of prematurity (ROP), which is categorized as the most fulminate type, was recognized. Both eyes were treated immediately, but dense laser photocoagulation with early re-treatment could not stop the progression to bilateral retinal detachment (Fig. 2). As for neuroimaging, cranial US revealed no abnormal findings for several days after birth. However, US obtained on the 14th day showed bilateral ventricular dilatation, and, thereafter, this finding spread extensively with cystic change. Brain MRI performed on the 217th day showed extensive reduction of cerebral hemisphere white matter with a large frontal cystic change (Fig. 3). Subsequently, intractable seizures began at the age of 18 months. At 2 years, the patient manifested severe psychomotor delay and marked growth retardation (weight was 4.5 kg).

### Cytogenetic analysis

Chromosomal analysis of peripheral blood lymphocytes by conventional routine G-banding (550 bands; 20 metaphase cells) showed an interstitial deletion ranging over two bands on the distal portion of the short arm of chromosome 4. The karyotype designation was 46,XY,del(4)(p15.3 p16). Moreover, it was confirmed by fluorescence in situ hybridization (FISH) analysis using a WHS microdeletion probe (LSI WHS, Vysis) that the deletion included WHSCR. Parental chromosome and FISH analyses showed normal chromosome complements.

## Discussion

WHS is caused by deletion of the WHSCR of chromosome 4p16 by one of several genetic mechanisms. About 75% of individuals with WHS have a *de novo* deletion of 4p16, and in 85% of *de novo* deletions, the origin of the deleted chromosome is paternal (Dallapiccola et al. 1993). About 12% have an unusual cytogenetic abnormality (such as ring 4). About 13% have deletion of 4p16 as the result of an inherited unbalanced chromosomal rearrangement from a parent with a balanced rearrangement, and in almost two-thirds of individuals with an inherited translocation, the mother carries the rearrangement (Bauer et al. 1985). In our case, the deletion was *de novo* from the cytogenetic analyses of both parents, and we informed the parents of this patient that the risk of recurrence in a future pregnancy would be negligible.

Structural brain abnormalities in WHS mainly include thinning of the corpus callosum in a few cases, with diffusely decreased white matter volume, which can be identified with prenatal US or MRI (Keersmaecker et al. 2002). In our case, the lateral ventricles had gradually enlarged after birth and extended to severe periventricular leukomalacia (PVL), although prenatal MRI and pre- and early postnatal US revealed normal brain anatomy. Therefore, these white matter lesions were supposed to be attributed to some perinatal hypoxic-ischemic event. In addition, white matter lesions in WHS may not be as severe as those found in our case, according to a recent study (Righini et al. 2007). PVL is the most common ischemic brain injury in premature infants, and is associated with perinatal events such as chorioamnionitis, prolonged rupture of membranes, asphyxia, and recurrent apnea. Generally, however, premature infants born at 31 weeks’ gestation rarely manifest such severe PVL as identified in our case. Ophthalmic findings in WHS are variable. As for retinal manifestations, foveal hypoplasia and chorioretinal colobomas are the only two conditions that have been reported to be associated with WHS (Wu-Chen et al. 2004), and there has been no report of a vasoproliferative retinal disorder or retinal detachment. On the other hand, ROP has occurred most frequently in premature infants. Generally, however, premature infants born at 31 weeks’ gestation rarely have the aggressive posterior ROP identified in our case without exposure to risks such as excessive oxygen and prolonged use of ventilators. As for genotype-phenotype correlations in WHS, some investigators have demonstrated that phenotype is partially or completely dependent on the size of the deletion (Wieczorek et al. 2000; Zollino et al. 2000), but others have concluded that the size of the deletion does not correlate with severity of clinical findings (Meloni et al. 2000). Therefore, we do not think that the brain and ocular findings in our case are associated with only the congenital anomaly or only prematurity. It is presumed that latent structural fragility, which may be associated with WHS, severely aggravated the brain and retinal lesions caused by prematurity.

Our case raises two important considerations about prenatal management of WHS: how to recognize it, and when to deliver. First, concerning the prenatal detection of WHS, it has been well known that infants exposed to early fetal malnutrition due to congenital abnormalities are symmetrically small. Prenatally, our case was recognized to have asymmetrical IUGR and was not highly suspected to have congenital abnormalities. However, recent data demonstrated that congenital abnormalities were found in both symmetric and asymmetric IUGR (David et al. 1995). Prenatal diagnosis of WHS may be difficult unless the fetus is karyotyped for severe IUGR that is diagnosed on routine US. An accurate diagnosis may also be missed based on genetic amniocentesis, because standard karyotyping based on routine G-banding during pregnancy was found to be normal in half of cases (Battaglia et al. 2001). FISH using a probe that includes the entire WHSCR detects a deletion in more than 95% of individuals with WHS (Battaglia et al. 2006). Recently, the possibility of recognizing the particular phenotype of WHS by prenatal US has been reported (Boog et al. 2004; Sase et al. 2005). The associated sonographic signs of a typical face, cystic cerebral lesions, midline fusion defects or bilateral renal hypoplasia, especially with a normal amniotic fluid volume, may help to refine specific indications for high-resolution banding and molecular analysis by FISH. According to some reports, however, once a prenatal diagnosis of WHS was confirmed, pregnancy was interrupted at the family’s request in most cases (Keersmaecker et al. 2002; Boog et al. 2004). As a practical consideration, children with WHS have many medical problems that are worrisome and difficult to manage, but this is not a fatal chromosomal anomaly like trisomy 13 or trisomy 18. Accurate information on the natural history of WHS is of paramount importance to obstetricians who assist families in making relevant decisions about possible interruption of pregnancy. The second concern is determination of the optimal timing of delivery. Previously, there was a general consensus on the management of IUGR that delivery was best delayed until term. With the current understanding and with advances in antenatal and neonatal assessment, preterm delivery tends to be indicated if the growth-restricted fetus demonstrates abnormal fetal function, which is often recognized as the absence of demonstrable fetal growth, because delay in delivery may increase hypoxia and affect brain development (Fang, 2005). On the other hand, in the case of a fetus with a chromosomal defect, the risks of prematurity must be weighed against the complications unique to IUGR and congenital dysmorphism if preterm delivery is indicated. In the context of IUGR, even if it is asymmetric, detailed US examination should be performed to determine the need for fetal karyotyping. Then, once the prenatal diagnosis of WHS is confirmed, the delivery should be delayed as long as possible to achieve fetal maturation unless a non-reassuring fetal state is evident.
